# Laplace approximation for conditional autoregressive models for spatial data of diseases

**DOI:** 10.1016/j.mex.2022.101872

**Published:** 2022-10-01

**Authors:** Guiming Wang

**Affiliations:** Department of Wildlife, Fisheries and Aquaculture, Mississippi State University, Mississippi State, Mississippi 39762, USA

**Keywords:** Bayesian statistics, disease risk assessment, maximum likelihood estimation, template model builder, WinBUGS

## Abstract

Conditional autoregressive (CAR) distributions are used to account for spatial autocorrelation in small areal or lattice data to assess the spatial risks of diseases. The intrinsic CAR (ICAR) distribution has been primarily used as the priori distribution of spatially autocorrelated random variables in the framework of Bayesian statistics. The posterior distributions of spatial variates and unknown parameters of Bayesian ICAR models are estimated with the Markov chain Monte Carlo (MCMC) methods or integrated nested Laplace approximation (INLA), which may suffer from failures in numeric convergence. This study used the Laplace approximation, a fast computational method available in software Template Model Builder (TMB), for the maximum likelihood estimation (MLEs) of the ICAR model parameters. This study used the TMB to integrate out the latent spatial variates for the fast computations of marginal likelihood functions. This study compared the runtime and performance among the TMB, MCMC, and INLA implementations with three case studies of human diseases in the United Kingdom and the United States. The MLEs of the ICAR model with TMB were similar to those by the MCMC and INLA methods. The TMB implementation was faster than the MCMC (up to 100–200 times) and INLA (nine times) models.

• This study built conditional autoregressive models in template model builder

• TMB implementation was 100-200 times faster than the MCMC method

• TMB implementation was also faster than Bayesian approximation with R INLA

## Graphical abstract

**Figure:**
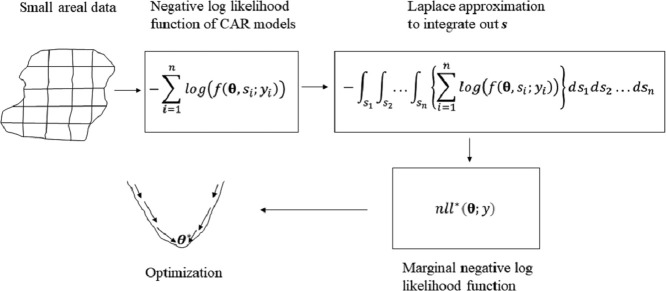

Specifications table

**Table:** 

Subject area;	Environmental Science
More specific subject area;	Applied spatial statistics for ecological risk assessments
Name of your method;	Laplace approximation for maximum likelihood estimation of CAR spatial statistical models
Name and reference of original method;	[1]. Spatial interaction and the statistical analysis of lattice systems. Journal of the Royal Statistical Society Series B 36: 192-236.
Resource availability;	The C++ codes, R code and data used in this work are available in the supplemental material

## Method details

Spatial autocorrelation is commonly found in geographically registered data. Ignoring spatial autocorrelation results in the bias in the parameter estimates of statistical models. For instance, disregarding spatial autocorrelation may lead to the missing of the disease risk hotspots and underestimate the variance of unknown parameters, misleading the statistical inferences of disease risks. Data on human and wildlife infectious disease are often collected as small areal data (i.e., observations in the neighboring polygons or in the grid cells of a lattice). Conditional autoregressive (CAR) distributions have been used to account for spatial autocorrelation in small areal data [1,2,10]. Conditional autoregressive models are the first-order Gaussian Markov Random Field (GMRF) models on a 2-dimensional plane [12,15]. The GMRF model assumes the multivariate normal distribution for a spatial variate and its first-order neighborhood with a sparse covariance matrix, which has majority of its elements being zero and only non-zero elements between the pairs of the neighboring cells (i.e., the pairwise Markov property). The latent spatial variates within the first-order spatial neighborhood are assumed to be independent, conditional on the sparse covariance matrix and the spatial neighborhood (the local Markov property) [1,15]. Thus, the conditional spatial independence can be used to account for the spatial autocorrelation in the small areal data [7]. The most popular CAR model for disease risk assessments is the intrinsic CAR (ICAR) model. The ICAR model is a special case of CAR models, of which the covariance matrix is simplified by omitting a parameter that is difficult to estimate (Eqn. 1 below) [1,2,8]. Consequently, the covariance matrix of the ICAR distribution is not invertible due to the simplification [1,14]. Therefore, the sum-to-zero constraint on spatially autocorrelated random variates has to be imposed to make the ICAR model identifiable [7,14]. This technicality creates some difficulties in the development of fast, reliable computational methods for the ICAR models, particularly in the frequentist framework [20].

In the framework of Bayesian statistics, the ICAR distribution is used as a prior distribution for spatially autocorrelated random variates in conjunction with the uniform prior from negative to positive infinity for the intercept or mean [7,14]. The posterior marginals of unknown parameters are proportional to the joint posterior distributions to a constant. The Markov chain Monte Carlo (MCMC) sampling is commonly used to estimate the posterior marginals of the unknown parameters of the ICAR model [10]. However, the MCMC chains for the ICAR model may suffer from failures in the numeric convergence on the WinBUGS platform, which is not rare in the Bayesian computation. Furthermore, the full Bayesian approach estimates the latent spatial variates using the MCMC methods, which increases the computational burdens. Alternatively, the R package INLA (Integrated Nested Laplace Approximation) includes different CAR models using approximate Bayesian computation (ABC) without the concern of the MCMC convergence [12]. The INLA approach uses the GMRF to represent the set of the latent parameters (including unknown parameters and linear predictors) of the generalized linear models and their extensions (i.e., model parameters, linear predictors, and latent spatial variates) of spatial and spatiotemporal statistical models [16]. The INLA first uses the Laplace approximation and the Bayes’ Rule to compute the joint marginal posteriors of model parameters and latent variables, respectively. Then the INLA numerically approximates the marginal posteriors of individual parameters or a latent variable by integrating out all other parameters or latent variables with the Laplace approximation (i.e., Laplace approximation in a nested formulation) [17]. However, the nested Laplace approximation can be slow and fail to converge numerically [13]. Compared to the MCMC and ABC implementations of ICAR models, fewer computationally efficient CAR models are available in the frequentist framework [20]. Therefore, there is a need for different computational methods for CAR models for small-areal spatial data. This study used the Laplace approximation within template model builder (TMB) to generate the marginal likelihood function of the ICAR models, and then maximize the marginal likelihood function to estimate the unknown parameters of the ICAR model [9]. The TMB does not directly estimate the latent spatial variates like its Bayesian counterpart; instead, the TMB uses a plug-in method to estimate latent variables reducing computational burdens [19]. In a recent study, Wang [11] implemented multinomial-Dirichlet (M-D) models in TMB, which ran a few hundred times faster than Bayesian M-D models in JAGS or Stan, a computer program for Bayesian statistics using the No-U-Turn sampler (NUTS) for the MCMC chains [5]. However, the performance of the TMB for small areal spatial data was unknown compared to the MCMC and ABC approaches.

This study implemented the ICAR model using the TMB, WinBUGS, and INLA to compare computational performances among the TMB, WinBUGS, and INLA implementations and demonstrate the computational efficiency and advantages of the TMB implementation over the MCMC and INLA methods. The ICAR model functions in the WinBUGS and INLA are ready to use. This study implemented the ICAR models using the TMB C++ template. We presented the mathematics and statistical expressions of the ICAR models below, which were used in the TMB implementation, for readers to understand and examine the TMB C++ codes attached in the supplemental material.

### The Besag intrinsic conditional autoregressive model

Besag [1] proposed CAR distributions for positive spatial autocorrelation in the lattice or the Dirichlet polygons. Let *s_i_* denote a random variate in the polygon *i* and *s_1_, s_2_, …, s_i-1_, s_i+1_,* …, $s_{n_{i}}$are $n_{i}$-1 the nearest or first-order neighboring polygons, of which each is directly bordered with polygon *i*. Intrinsic CAR distributions assume *s_i_* following a conditional normal distribution:

(1) $$s_{i}|s_{-i}∼N\left(\mu_{i}+\sum_{j=1}^{n_{i}}\beta_{ij}\left(s_{i}-\mu_{i}\right),\sigma_{i}^{2}\right),$$

where *μ_i_* is the mean of the spatial neighboring structure ***s***; $s_{-i}$ in the right side of the symbol “|” is the *n_i_*-1 first-order neighbors of polygon *i*, indicating a conditional distribution of *s_i_* on its nearest neighbors; $\sigma_{i}^{2}=\sigma_{s}^{2}/n_{i}$; and $\sigma_{s}^{2}$ is the variance of ***s***. In the ICAR distributions, $\beta_{ij}$ is set to weight $W_{ij}$, where $W_{ij}=1\;$if i≠j; otherwise, $W_{ij}=0$. Intrinsic CAR models regress *s_i_* on the values of its directly bordering neighbors, providing a smoothing estimate of spatial effects within the nearest neighborhood.

### Intrinsic conditional autoregressive models for the spatial structure in Poisson count

This study focused on disease risk assessments using the count (*y*) of infection cases with the following model structure:

(2) $$\begin{array}{ccc} y_{i} & ∼ & Pois\left(\lambda_{i}E_{i}\right),\\ \eta_{i} & = & \log\left(E_{i}\right)+\log\left(\lambda_{i}\right),\\ \log\left(\lambda_{i}\right) & = & \beta_{0}+\sum_{j=1}^{p}\beta_{j}x_{ij}+s_{i},\;\;\;i=1,2,...,N,\; \end{array}$$

where *E_i_* is the offset of polygon *i*; $\lambda_{i}$ is the relative risk of diseases; *β_0_* is intercept; *β_j_* is the coefficient of covariate *x_ij_* in polygon *i*; and *s_i_* is the spatially structured variate of polygon *i*, which follows an ICAR distribution (Eqn. 1).

### The implementation of the ICAR models in template model builder

Template model builder is a C++ based program for implementing hierarchical or multi-level statistical models using MLE methods [9]. Template model builder uses automatic differentiation (AD) to calculate the first- and second-order derivatives fast and accurately, which substantially speeds up the optimization of the Laplace approximation [18]. The TMB package also takes the maximal computational advantage of the sparse covariance matrix for the fast maximization of model likelihood functions. The negative log likelihood function of Eqs 2 can also be implemented in the matrix form as the following [7]:

(3) $$\begin{array}{ccc} p\left(s|\tau \right) & \propto & \exp\left(-\frac{1}{2\tau^{2}}s^{\prime }\left(D_{w}-W\right)s\right)\\ nll & = & -\sum_{i=1}^{n}\left\{y_{i}\left(\log\left(E_{i}\right)+\left(\beta_{0}+\sum_{j=1}^{p}\beta_{j}x_{ij}+s_{i}\right)\right)-\left(E_{i}\exp\left(\beta_{0}+\sum_{j=1}^{p}\beta_{j}x_{ij}+s_{i}\right)\right)\right\}\\ & & +\;\frac{1}{2\tau^{2}}s^{\prime }\left(D_{w}-W\right)s, \end{array}$$

where **s** is a vector spatial variate *s_1_, …, s_n_*; **s**’ is transposed **s; W** is the spatial weight matrix with its element ij being equal to 1 if polygon *j* is a first-order neighbor of polygon *i*; and **D**_w_ is a diagonal matrix with its diagonal elements (D_ii_) being equal to the number of first-order neighbors of polygon *i* (*i* ≠ *j*) or the row sum of matrix **W**. The terms $\log(y_{i}!)$ is omitted from Eqn 3 as well. Matrix **Q** = **D**_w_−**W** is singular, not invertible; thus, instead of inverting **Q** to produce a covariance matrix for multivariate normal distributions, negative log joint probability density functions (i.e., *nll*) in Eqn 3 was integrated with regard to (w.r.t) random variable vector **s** to produce a negative log marginal likelihood function (nll*) by the Laplace approximation in TMB,

(4) $$nll^{*}=\int \ldots \int nll\left(\theta ,s;y\right)ds,\;\;\;\;\;$$

where **θ** is the unknown parameter vector including *β_0_, β_j_*, and τ; **s** is a vector of spatial variate *s_1_, s_2_*, …, *s_n_*. The negative log likelihood functions of Eqs 3-4 were implemented in the TMB C++ template and then were minimized by the R function optim(). The convergence was checked with the output convergence indicator of optim() and the maximum gradient (derivative) of the log joint likelihood (≈0). The TMB C++ code was available in the supplemental material.

### The MCMC implementation of ICAR models in WINBUGS

The Bayesian estimation of the unknown parameters of ICAR model with the MCMC approach was implemented in WinBUGS. The WINBUGS codes were modified from [22] and is available in the supplemental material.

### The integrated nested Laplace approximation of ICAR models

The INLA method uses Laplace approximation to estimate the posterior marginals of unknown parameters, latent variables, and hyperparameters, whereas the TMB method uses Laplace approximation to integrate out latent variables to estimate the marginal likelihood functions without any prior distributions. The INLA assumes that the unknown parameter and linear predictors are latent random variables following GMRF distributions. So these latent variables have multivariate normal or nearly normal distributions with the means of zeros and sparse precision matrices. Instead of estimating multivariate Gaussian distributions, INLA applies Laplace approximation to estimate the posterior marginals of individual unknown parameters (including the hyperparameters of the MGRF precision matrices) or latent variables, respectively, in a nested structure. The review of the INLA estimation procedures goes beyond the scope of this work. Interested readers can find the statistical details in Rue et al. [17]. The RINLA code was modified from [21] for each case study.

**Fig. 1:**
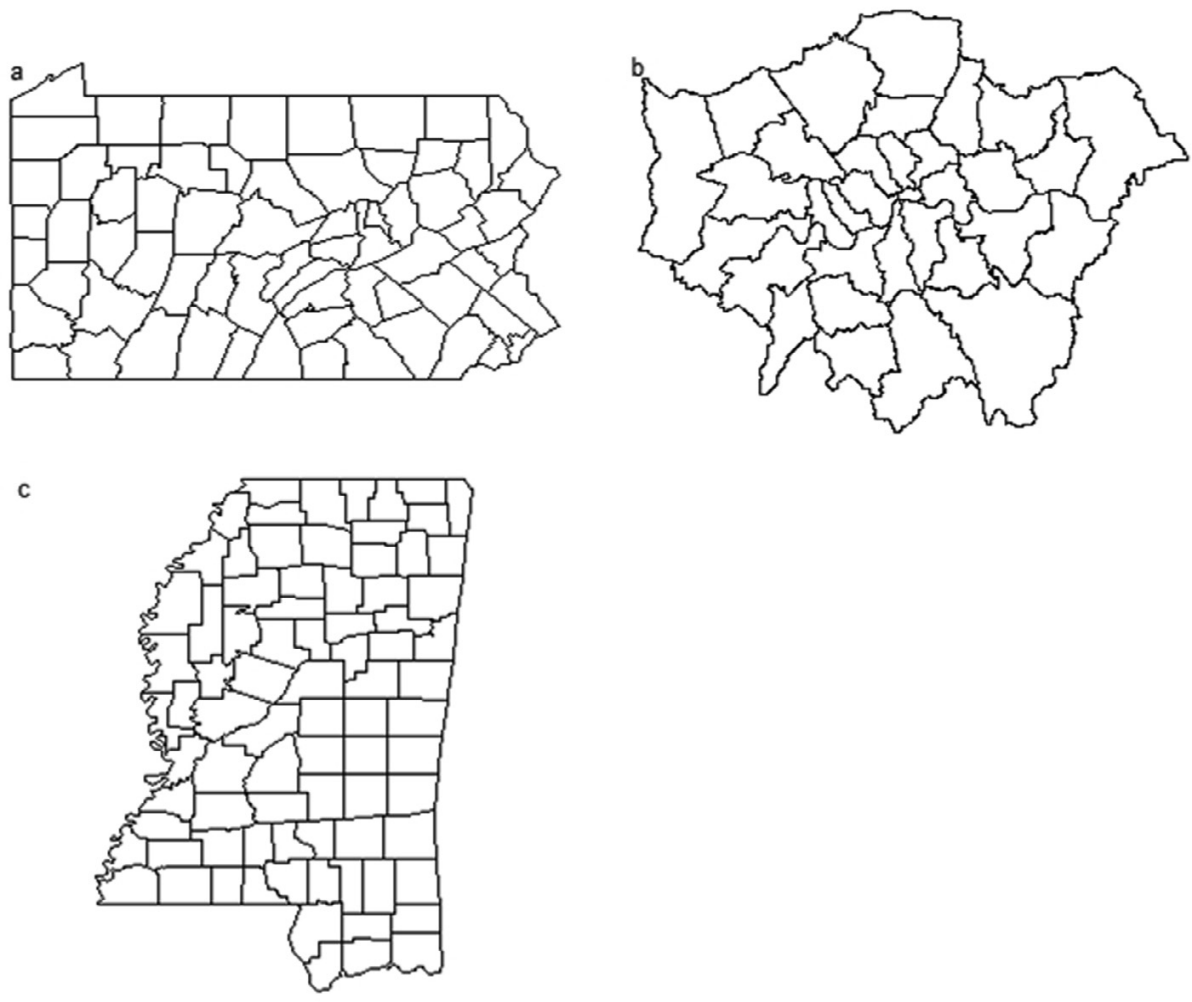
(a) Polygons of 67 counties of the State of Pennsylvania, the United States. A polygon represents the administrative boundary of a county. (b) Polygons of 32 boroughs, London, the United Kingdon. Each polygon represents the administrative boundary of a borough. Human suicide data did not include those from the City of London. The shapefile and data were obtained from Blangiardo and Cameletti [3]. (c) Polygons of 82 counties of the State of Mississippi, the United States. A polygon represents the administrative boundary of a county.

This study used three case studies of human diseases in the United Kingdom (UK) and the United States (US), which were published in the literature [4,13,22]. The response variables are the number of human suicides or the count of human disease incidents reported in an administrative unit (i.e., small areal data), which is assumed to have Poisson distributions. Each administrative unit has an irregular boundary polygon, which has various numbers of adjacent polygons (i.e., the first-order neighbors; Fig. 1). The boundary polygons are often saved as a GIS shapefile. The case study of the human infections of the West Nile virus in Mississippi was provided in the file “ms_human_wv_data.R”. The data will be loaded into the R environment by running the R code line ‘source(“ms_human_wv_data.R”)’. The GIS shapefile of the State of Mississippi boundary with the boundaries of 82 counties was saved in “mississippi_county.shp” available in the supplemental material. The R codes for loading and preparing data and running the TMB, WinBUGS, and INLA models are available in the file “ms_west_nile_final.R” in the supplemental material.

### Data on Pennsylvania lung cancer in 2002

The number of lung cancer cases was compiled for each of the 67 counties of Pennsylvania, the US (Fig. 1a) in 2002. The number of expected lung cancer cases (*E*) was calculated by multiplying the rate of lung cancer and population size using the function expected() within the R package SpatialEpi [13]. Data on the number of lung cancer cases, expected number of lung cancer cases, and the shapefile of the state and county boundaries of Pennsylvania were obtained from the R package SpatialEpi [13]. The Besag ICAR model, $y_{i}∼Pois\left(E_{i}\lambda_{i}\right)$, $log\left(\lambda_{i}\right)=\beta_{0}+s_{i}$, *i* = 1, 2, …, 67, was fit to the lung cancer data with TMB, R INLA, and WinBUGS, respectively. Symbol $\beta_{0}$ is the intercept or mean and $s_{i}$ is spatially correlated variate in polygon i.

### Data on suicides in London

Congdon [6] studied suicide mortality in the 32 boroughs of London, the UK from 1989 to 1993 (Fig. 1b). The number of suicides, the expected number of suicides (*E*), and the shapefile of the boundaries of the 32 boroughs were obtained from [3]. The Besag ICAR model, $y_{i}∼Pois\left(E_{i}\lambda_{i}\right)$, $log\left(\lambda_{i}\right)=\beta_{0}+\beta_{1}x_{i1}+\beta_{2}x_{i2}+s_{i}$, *i* = 1, 2, …, 32, was fit to the suicide data with TMB, R INLA, and WinBUGS, respectively. The notation *β_j_* (*j* = 0, 1, 2) is regression coefficient, *x_i1_* is social deprivation, *x_i2_* is social fragmentation, and *s_i_* is spatially correlated variates.

### Data on human West Nile virus infection cases in Mississippi in 2002

Wang et al. [22] analyzed data on the human West Nile virus incidences in the 82 counties (Fig. 1c) of Mississippi, the US in 2002 using the ICAR model within WinBUGS. The expected number of human West Nile virus infections was estimated for each county by multiplying county human population size of the US 2000 Census with the 2002 US national human incidence rate. The Besag ICAR model, $y_{i}∼Pois\left(E_{i}\lambda_{i}\right)$, $log\left(\lambda_{i}\right)=\beta_{0}+s_{i}$, *i* = 1, 2, …, 82, was fit to the human West Nile virus infection data with TMB, R INLA, and WinBUGS, respectively. Symbol $\beta_{0}$ is the intercept or mean and $s_{i}$ is spatially correlated variate in polygon i.

### Comparisons of model performances and runtime among the TMB, MCMC, and INLA implementations

All three estimation methods produced similar estimates of the intercept for the 2002 lung cancer data of Pennsylvania (Table 1). The estimates of both the mean and standard error only differed at the third decimal place. The MLE estimates of the variance of spatially autocorrelative variates by TMB were slightly lower than those by the MCMC with WinBUGS and Bayesian approximation with R INLA (0.13 vs. 0.14, Table 1). Template model builder produced similar estimates of regression coefficients with those by the MCMC with WinBUGS for the suicide data in London (Table 2). Likewise, the estimates of intercept and spatial variance of the ICAR model for the human West Nile virus infections of Mississippi in 2002 were similar between the MLE and Bayesian estimates (Tables 3). The TMB models was 9 times faster that the INLA models and >100 times faster with the MCMC on the WinBUGS (Table 1, Table 2, Table 3).

**Table 1:** Parameter estimates of intrinsic conditional autoregressive models for the number of lung cancer cases of 67 counties in the State of Pennsylvania, the United States in 2002 [13]. Parameter τ is the variance of spatially autocorrelative variates. The parameters were estimated by maximum likelihood method in template model builder (TMB), Markov chain Monte Carlo methods in WinBUGS (BUGS), and Bayesian approximation in R package INLA, respectively. Runtime is the computer elapse time during the execution of each program.

Coefficient	TMB	BUGS	INLA
Intercept	-0.050 (0.015)	-0.051 (0.014)	-0.050 (0.016)
Spatial τ	0.133 (0.031)	0.142 (0.032)	0.142 (0.032)
Runtime (s)	0.05	12.86	0.87

**Table 2:** Parameter estimates of intrinsic conditional autoregressive models for the number of human suicides in 32 boroughs in London, the United Kingdom from 1988 to 1992 [6]. Parameter τ is the variance of spatially autocorrelative variates. The parameters were estimated by maximum likelihood method in template model builder (TMB), Markov chain Monte Carlo methods in WinBUGS (BUGS), and Bayesian approximation in R package INLA, respectively. Runtime is the computer elapse time during the execution of each program.

Coefficient	TMB	BUGS	INLA
Intercept	0.056 (0.017)	0.059 (0.016)	0.058 (0.016)
*β_1_* (social deprivation)	0.115 (0.022)	0.115 (0.022)	0.114 (0.024)
*β_2_* (social fragmentation)	0.175 (0.023)	0.175 (0.024)	0.175 (0.025)
Spatial *τ*	0.144 (0.050)	0.142 (0.049)	0.164 (0.055)
Runtime (s)	0.14	22.77	1.11

**Table 3:** Parameter estimates of intrinsic conditional autoregressive models for the number of human West Nile virus cases in 82 counties in the State of Mississippi, the United States in 2002 [22]. Parameter τ is the variance of spatially autocorrelative variates. The parameters were estimated by maximum likelihood method in template model builder (TMB), Markov chain Monte Carlo methods in WinBUGS (BUGS), and Bayesian approximation in R package INLA, respectively. Runtime is the computer elapse time during the execution of each program.

Coefficient	TMB	BUGS	INLA
Intercept	-0.196 (0.123)	-0.195 (0.122)	-0.205 (0.123)
Spatial *τ*	1.170 (0.264)	1.155 (0.255)	1.195 (0.257)
Runtime (s)	0.10	14.56	0.92

### Advantages of the template model builder for ICAR models

This study has demonstrated the feasibility to use the Laplace approximation of TMB to integrate out spatial variates for the MLE of the ICAR model. Template model builder can detect the sparsity of covariance matrices and use the sparse matrix to enhance and speed up the maximization of likelihood functions [9].

The fast runtime of TMB, as shown in this study, can help us develop the ICAR model in multiple ways. First, TMB helps with the development and variable selection of the ICAR models, iterating through various models of different structures in a reasonable timeframe. Second, TMB is free of the MCMC convergence issue. Bayesian model parameterization, such as the choice of the prior distributions and initial values of model parameters, can facilitate the convergence of the MCMC chains. The results of the ICAR model with TMB may provide information for the prior choice and initial values of the full Bayesian CAR models to facilitate the MCMC convergence [11].

## CRediT authorship contribution statement

**Guiming Wang:** Conceptualization, Methodology, Software, Investigation, Writing – original draft, Writing – review & editing.

## Declaration of interests

The authors declare that they have no known competing financial interests or personal relationships that could have appeared to influence the work reported in this paper.

## Data Availability

I have shared files as attachments.

## Appendix Supplementary materials

Image, application 1
